# Antivirulence and avirulence genes in human pathogenic fungi

**DOI:** 10.1080/21505594.2019.1688753

**Published:** 2019-11-11

**Authors:** Sofía Siscar-Lewin, Bernhard Hube, Sascha Brunke

**Affiliations:** a Department of Microbial Pathogenicity Mechanisms, Hans Knoell Institute, Jena, Germany; b Institute of Microbiology, Friedrich Schiller University, Jena, Germany

**Keywords:** Fungal virulence, antivirulence, avirulence, host-pathogen interactions, evolution of virulence

## Abstract

Opportunistic commensal and environmental fungi can cause superficial to systemic diseases in humans. But how did these pathogens adapt to infect us and how does host-pathogen co-evolution shape their virulence potential? During evolution toward pathogenicity, not only do microorganisms gain virulence genes, but they also tend to lose non-adaptive genes in the host niche. Additionally, virulence factors can become detrimental during infection when they trigger host recognition. The loss of non-adaptive genes as well as the loss of the virulence potential of genes by adaptations to the host has been investigated in pathogenic bacteria and phytopathogenic fungi, where they are known as antivirulence and avirulence genes, respectively. However, these concepts are nearly unknown in the field of pathogenic fungi of humans. We think that this unnecessarily limits our view of human-fungal interplay, and that much could be learned if we applied a similar framework to aspects of these interactions. In this review, we, therefore, define and adapt the concepts of antivirulence and avirulence genes for human pathogenic fungi. We provide examples for analogies to antivirulence genes of bacterial pathogens and to avirulence genes of phytopathogenic fungi. Introducing these terms to the field of pathogenic fungi of humans can help to better comprehend the emergence and evolution of fungal virulence and disease.

## Introduction

Diseases caused by pathogenic fungi are still frequently underestimated [1] but are more and more recognized as an important threat especially to immunocompromised populations. Currently, treatment of fungal infections in humans is limited to the use of a few classes of antifungal drugs [2]. Understanding the pathobiology of these fungi is essential to develop novel therapeutic approaches to extend our options to treat fungal infections. Diseases caused by fungi come in many forms: Dermatophytes affect approximately one fifth of the world population but are restricted to cause infections of skin, hairs, and nails. In contrast, *Candida, Aspergillus, Cryptococcus, Coccidioides*, *Pneumocystis* species, and *Histoplasma capsulatum*, are among the most important fungi that are able to cause diseases ranging from superficial to systemic [3]. Whereas they mainly affect immunocompromised hosts, a few environmental species are primary pathogens and can also cause disease in healthy individuals [3]. *Candida* spp. are among a small group of fungal species that are thought to have been commensal members of our microbiota for much of human evolution [3–7]. Likely because of this coevolution, these species have developed an impressive range of adaptations to the human environment, which allow the fungus to obtain nutrients, survive to host immunity, and withstand stress conditions within the human host – all of which is not only required for commensalism, but also a pre-requisite for pathogenicity [8–12].

Other human pathogenic fungi, although having evolved as saprophytes in the environment or in close relationships with birds and bats (like *Cryptococcus neoformans* and *H. capsulatum*) or rodents (like *Coccidioides* species) often exhibit infection strategies strikingly similar to the human commensal *Candida* species, from immune evasion to hydrolytic enzymes and toxins [13,14]. In fact, these convergent evolved strategies frequently resemble the mechanisms used to resist environmental phagocytes, like amoebae [15]. It has been suggested that an “environmental virulence school” allowed them to become successful human pathogens [3], as the same mechanisms allow them to resist, shield themselves, counteract and manipulate host immune responses [13]. Such training grounds for host interactions, commensal and environmental, might thereby explain the appearance of virulence factor genes in human pathogenic fungi.

However, during the evolution of pathogenicity, fungi must also shed certain genes which are involved in “energy wasting” processes and have no selective advantage in the host or even trigger detrimental host responses. Such non-adaptive genes, known also as antivirulence genes, are very well described in bacterial pathogens [16]. In fact, the evolution toward pathogenicity of some very important infectious microorganism, such as *Yersinia pestis, Shigella* or *Francisella tularensis* (subsp. *tularensis* and *holarctica*), has been characterized by loss of genes [16,17]. Moreover, not all virulence factors are solely beneficial to the pathogen during interactions with the host. Virulence factors and their associated damage can also make the pathogen “visible” to the host’s immune surveillance and therefore become disadvantageous [18,19]. Such factors have been studied in pathogenic fungi of plants, which are able to trigger a hypersensitive immune response in the infected plant and thereby promote plant resistance [20]. This interaction renders the pathogen avirulent, and genes encoding these effectors are consequently known as avirulence genes [20,21]. We propose that similar events take place in human-fungal interactions, and that we can analogously identify antivirulence and avirulence genes in fungal pathogens of humans. Thinking in these terms may help us to better understand the host-pathogen interplay, and eventually help us in the search for new fungal therapeutic targets to combat fungal infections.

## The concept of antivirulence gene in human pathogenic fungi

To define antivirulence in fungi, we need first to have – for this review – a working definition of virulence for these organisms. It is now textbook knowledge that, while pathogenicity refers to the ability of a microbe to cause damage in a host *per se*, virulence is defined by the degree of damage the microbe can elicit (Box 1). This damage manifests as the interruption of normal host function (and usually tissue structure) at any of the cellular, tissue or organ levels, which as clinical manifestation is called disease [22,23].

**Box 1.** Definitions of concepts.

**Pathogenicity**: the ability of a microbe to cause damage to a host.
**Virulence**: the degree of damage a microbe can elicit. This damage manifests as the interruption of normal host function (and usually tissue structure) at any of the cellular, tissue or organ levels, which as clinical manifestations is called disease.
**Virulence factor**: a microbial attribute that causes damage to a host, either by direct action or indirectly by the host response or by allowing growth and survival when facing the host reaction (the latter is often also called a virulence determinant). The deletion of a virulence factor gene usually reduces virulence.
**Antivirulence factor**: a microbial attribute that reduces the direct or indirect damage to the human host. The gene that encodes an antivirulence factor is an **antivirulence gene**, and its deletion or loss leads to an increased virulence.
**Avirulence factor**: a virulence factor that is recognized by a host specific receptor, which triggers a host immuno response that concludes with the pathogen’s virulence. The gene that encodes an avirulence factor is an **avirulence gene**.

**Figure 1.**
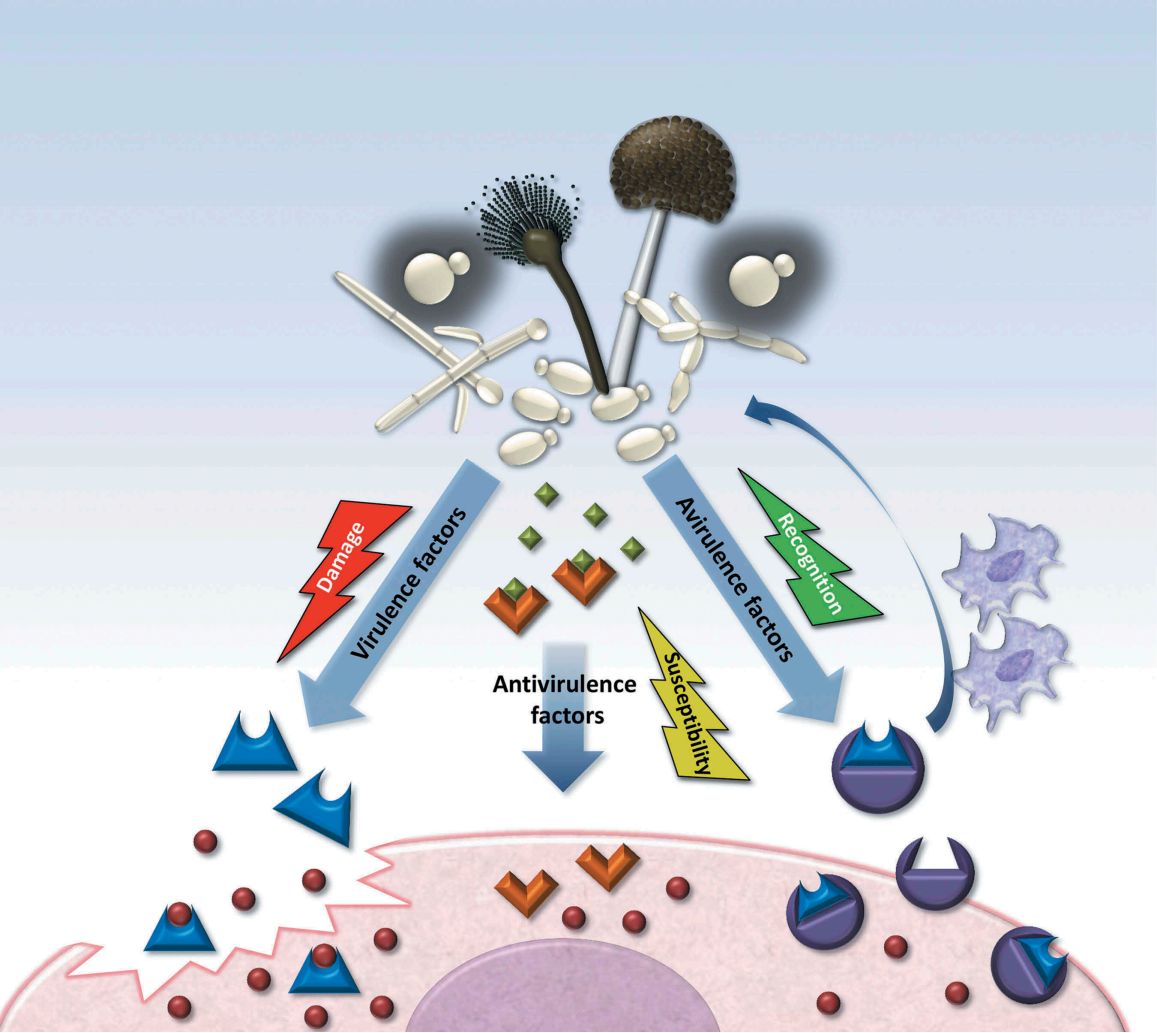
Illustration of virulence, antivirulence, and avirulence factors and their adaptive consequences within the host. Fungal factors expressed during host–pathogen interactions can lead to three different outcomes. From the pathogen’s perspective, a virulence factor (blue form) can be advantageous to overcome the host immune barrier, invade, or withstand stress conditions during infection. An antivirulence factor, in contrast, might be advantageous outside the host (green squares), but has a detrimental effect within the host, since it lowers the pathogen’s fitness, immune evasion ability or stress resistance. Lastly, a potential virulence factor can lose its function and become detrimental to the pathogen when the host develops specific receptors (purple form). If these recognize the factor or its action in the host, it can trigger an (immune) response that stops the progression of infection and turns the virulence factor into an avirulence factor.

Virulence itself can derive from direct action of the microbe, and classically virulence factors are defined as properties of the pathogen that, when deleted, impair their damage potential in the host but not their general viability (which is supported by virulence-independent, broad physiological factors) [23]. Examples of such virulence factors are the capacity to attach to or invade into host tissue (or both), as well as avoidance of host detection, inhibition of phagocytosis, and intracellular survival [24]. Even without dedicated virulence factors, microbial growth and persistence within the host can eventually induce damage through the host inflammatory response, and the virulence of some organisms is intrinsically linked to the ability of inducing a host inflammatory response that results in tissue damage [18,19,25]. On the other hand, even microbial virulence factors can only affect susceptible hosts, as exemplified by the many fungal pathogens which as opportunists do rarely affect an otherwise healthy host [3]. Therefore, the virulence of a microbe is evidently not solely dependent on microbial attributes but is rather determined by all of the host–microbe interactions. This requires us to extend on the previous concept of virulence, which explicitly excluded “physiological factors” that are essential for microbial growth. Such a more inclusive virulence factor definition then includes all microbial attributes that mediate host damage (Figure 1): from those essential for invasion to those essential for microbial growth within the host and a potential self-damaging host reaction [24]. Consequently, attributes that actively impair microbial fitness within the host or trigger an appropriate (i.e. not self-damaging) host recognition and response would be detrimental to the pathogen.

How virulence emerged across the human pathogenic fungi is still far from understood. One strategy to investigate the emergence of pathogenicity focusses on the comparison of genomes from the most closely related nonpathogenic and pathogenic species. This includes the study of past genomic re-arrangements, lineage-specific gene duplications, mutations and indicators of positive selection. For *Candida* species, the main mechanisms identified so far to promote pathogenicity are total or partial chromosomal rearrangements, gene duplication and loss, gene family expansion, and inter-species hybridization [26]. In *A. fumigatus*, gene duplication and diversification in genomic islands is a known mechanism to acquire novel genes, which are absent in the nonpathogenic relatives and mainly encode secondary metabolites, such as mycotoxins [27,28]. Thus, these gene factories might become the source of new virulence factors in *A. fumigatus*. Clear examples of evolution toward pathogenicity are found in the genomes of different *Candida* species. It has been shown that genes encoding virulence-associated adhesins, like the *ALS* and *EPA* families of *C. albicans* and *C. glabrata*, respectively, or hydrolases like the *C. albicans SAP* or *LIP* families multiplicated in these pathogenic species [29,30]. In contrast, their loss has occurred in related yeasts: *C. dubliniensis* has lost, for example, *ALS3*, an important virulence gene in *C. albicans* [28]. Similarly, the nonpathogenic relative of *C. glabrata, Nakaseomyces delphensis*, has one single copy of the *EPA* genes whereas *C. glabrata* possesses 18 [29]. Finally, the causative agents of valley fever, *Coccidioides* spp. (*Coccidioides immitis* and *Coccidioides posadasii*), show enrichment in keratinase-encoding genes, a sign of their close association with keratin-rich animals during the evolution from a plant-saprophytic fungus to an animal pathogen [31].

For a microbe in contact with a host, the lack of nutrients, generally harsh environmental conditions and the need to evade immune defenses [12,14,32] can exert selective pressures toward the evolution of a virulent phenotype. Positively, this can be the acquisition of genes that encode virulence factors by gene transfer [33–35] or mutations as described above, but the loss of certain genes has been recognized more and more as an important event for the emergence of virulence in bacterial species [16,17]. These antivirulence genes (Figure 1), whose expression is largely or absolutely incompatible with an at least transient pathogenic lifestyle, are classically present and active in the genomes of nonpathogenic antecessor, but become pseudogenes or lost in pathogenic species [16]. Bliven et al. explicitly exclude genes that are active in the majority of wild-type strains of a species, but inactive in virulent strains, and call these regulators or suppressors of virulence instead. Per definition, the insertion of active antivirulence genes into derived pathogenic strains leads to a decrease in their virulence. Examples have been found in bacterial pathogens, such as *Shigella, Salmonella*, and *Yersinia* species: In contrast to its nonpathogenic antecessor *Escherichia coli, Shigella* species have lost the ability to synthesize *de novo* nicotinic acid (NAD) by inactivation of the genes *nadA* and *nadB* [36, 37]. It was shown that the pathway intermediate quinolinic acid inhibits the type III secretion system of *Shigella* spp., and thus its virulence. *Shigella* spp. instead imports exogenous nicotinic acid, and the introduction of the biosynthesis genes reduces their virulence – marking these genes as antivirulent. *Yersinia pestis*, in contrast to its milder enteric human pathogen antecessor *Y. pseudotuberculosis*, has lost metabolic and motility associated genetic loci for a successful colonization of the mammalian gastrointestinal tract and become a systemic pathogen that invades the lymphatic system. In fact, the gene loss experienced by *Y. pestis* comprises a considerably larger proportion of the genome than what has been acquired by gene gain events in the pathogenic *Y. pestis* lineage [38].

Unlike in pathogenic bacteria, to our knowledge no antivirulence genes have been explicitly named in human pathogenic fungi. However, we can find examples of pseudogenization and loss of genes accompanying the evolution toward both commensalism and pathogenicity in fungi [28]. In addition, hypervirulence as a result of experimental gene inactivation is frequently observed [39]. This indicates that loss of function is a possible evolutionary trajectory to increased virulence also in the human host. With this background, we will now look into possible antivirulence genes in pathogenic fungi. If we follow the very strict definition of antivirulence genes from Bliven and Maurelli [16], which requires both, avirulent antecessors and virulent descendant species, we would have to exclude from our investigation those genes that are absent or inactive in virulent strains, but active in nonpathogenic “wild type” strains of the same species. However, this makes antivirulence a property of the gene which is mainly dependent on the definition of species and the classification of wild types *vs*. derived strains. For the sake of this review, we will therefore include examples of genes which exert their antivirulent properties in nonpathogenic strains, but whose loss can be associated with a significant increase in virulence in clinical isolates or experimentally generated mutants. We thereby unlink the genes’ property from phylogenetic and epidemiological considerations.

## Potential antivirulence genes of human pathogenic fungi

### Antivirulence genes identified from pathogen evolution

The adaptation of a microorganism to a new environment requires the loss of non-adaptive genes in order to optimize the energy expenses in the new niche. *C. glabrata* has lost several metabolic pathway genes compared to the generally nonpathogenic yeast *Saccharomyces cerevisiae* [40]. These losses include genes of the galactose metabolism, nitrogen metabolism, and sulfur metabolism; their loss may have contributed *C. glabrata*’s adaptation to the human gastrointestinal tract [28,41,42]. Interestingly, one classical example is again connected to nicotinic acid, paralleling *Shigella* spp.: *C. glabrata* has lost its ability for *de novo* nicotinamide adenine dinucleotide (NAD^+^) biosynthesis and requires external nicotinic acid or niacin as precursors [43]. This auxotrophy allows it to detect the low niacin levels in the urinary tract and regulate, *via* lack of NAD^+^-dependent histone de-acetylation, the expression of its virulence-associated Epa adhesins [44]. In connection to this, in a limited NAD environment, the epigenetic regulator Hts1 is inactivated and triggers derepression of genes involved in oxidative stress and fluconazole resistance, themselves major pathogenic traits of *C. glabrata* [45]. Thus, a gene loss enables the fungus to correctly detect the host environment and commence a virulence-associated genetic program.

Other clear examples are found in large and often redundant gene families of the environmentally acquired dimorphic pathogens of mammals, *Coccidioides* spp. and *Histoplasma capsulatum*. The first, along with the duplication of genes thought to be adaptive in the animal host [31], shows a reduction in genes encoding plant cell wall degrading enzymes, such as cellulases, cutinases, tannases, and pectinesterases, in stark contrast, e.g. to plant saprophyte *Aspergillus* species. *H. capsulatum* has experienced a similar reduction of plant matter-degrading enzymes during the evolution toward an animal pathogenic phenotype [31]. It requires no large leap of the imagination to assume the replication and expression of such genes to be detrimental in the human host, where they would lead to unnecessary energy expenditure and become potential immune-recognition targets within the host.

### Antivirulence genes identified experimentally in host interactions

In laboratory evolution experiments, *C. glabrata* was shown to increase its virulence during long-term exposure to macrophages. During *in vitro* adaptation to the phagocytes, a single nucleotide mutation likely rendered a chitin synthase without function. The resultant change in growth morphology was associated with a transient increase in virulence and organ burden [46]. Moreover, in *C. neoformans*, the gene *ALL1*, involved in capsule formation, was shown to be downregulated during phenotypic switching to the so-called hypervirulent mucoid colony (MC) variant, which happens during chronical cryptococcal infections. This switching elicits damage-promoting inflammation and can lead to death of the host [47]. Jain et al. investigated the involvement of *ALL1* downregulation in the increased virulence of the MC variant. A knock-out mutant of *ALL1* was observed to lead to an ineffective immune response, failure to clear the pathogen, and decreased survival in animal models without any other impairment in fitness in the host environment [48]. Loss of *ALL1* influences capsule polysaccharides, which inhibit phagocytosis and impairs cell-mediated immune response. Furthermore, a H99L strain of *C. neoformans*, which was obtained by successive laboratory passages of the reference strain H99, shows inactivating mutations in the SAGA-associated factor gene *SGF29*. As a result, there is a reduction in histone acetylation and increased melanization. This was found to be associated with increased virulence in animal models and in addition, loss-of-function mutations in this gene were found in clinical isolates from patients with prevalent infections [49]. These examples show how spontaneous inactivation of certain genes can increase pathogen fitness and virulence during fungal infections.

In *C. albicans* and *C. glabrata*, cellular respiration affects host–pathogen interactions. In 2007, Cheng et al. [50] performed five serial passages of *C. albicans* through murine spleens by intravenous inoculation. They recovered a mutant with uncoupled oxidative phosphorylation, which was resistant to phagocytosis by neutrophils and macrophages. In long-term infections, this strain showed increased persistence and higher fungal burden in mice. In the case of *C. glabrata*, a clinical isolate that, as *petite* mutant, lacks fully functional mitochondria showed increased tissue burden in murine models compared to respiration-competent strains [51]. Thus, genes involved in respiration may be considered potential antivirulence genes in *Candida* species, which by inactivation can lead to increased fitness during infection.

### Antivirulence genes identified through knock-out mutations

In 2016 Brown et al. [39] reviewed examples of single genetic mutations that cause hypervirulence, which are collected in the pathogen–host interaction database (www.PHI-base.org). Seventeen examples were found among fungal pathogens of humans and plants, and currently, more than 20 potential antivirulence genes have been identified in important opportunistic pathogens: *A. fumigatus, C. albicans, C. glabrata*, and *C. neoformans*. Many of these genes are involved in cell wall morphogenesis and responses to stress that are frequently connected to immune evasion and stress resistance. In 2005, Tsitsigiannis et al. described a triple-deletion mutant of *A. fumigatus* lacking the genes *ppoA, ppoB*, and *ppoC* [52]. These encode fatty acid oxygenases required for the biosynthesis of oleic and linoleic acid-derived oxylipins, which in turn coordinate sexual and asexual sporulation [53]. The mutant was hypervirulent in a murine model of invasive pulmonary aspergillosis and showed increased tolerance to H_2_O_2_ stress. The authors suggest an oxylipin-mediated cross talk that induces host defenses against the development of pulmonary and invasive aspergillosis. In *C. neoformans*, the regulator of G protein signaling, Crg1 is a key regulator of pheromone-responsive mating. A *CRG1* mutant shows largely increased virulence in the prevalent and clinically important MATα strains of the fungus: Mouse survival time after infection was shortened by 40%, and the lethal dose was 100-fold lower than that of wild-type strains. Here, the activation of mating due to the *CRG1* deletion may have caused the upregulation of the Ste12 pathway that promotes melanin formation. Melanin in turn can increase stress tolerance and fungal survival, or alter the host immune response, thereby increasing virulence [54]. In the yeast *C. albicans*, a mutant lacking the cell wall protein Pir32 was found to be hyperfilamentous and hypervirulent, with increased resistance to different stressors [55]. The authors suggest that the lack of Pir32 on the cell surface was compensated by an increased chitin content, which is known to promote antifungal resistance [56–58]. Thus, inactivation of genes with roles in cell wall biosynthesis in different species led to more adaptive and virulent phenotypes in experimental infections. Often, these seem to be mediated by compensatory stress responses of the fungal cell, which are induced by the lack of the gene in established networks. Such pre-stressed cells can be more resilient when facing the host immune response and therefore over-compensate the detrimental effect of the gene loss in the context of the host.

Other potential antivirulence genes have been identified counter-intuitively by knocking out genes which were expected to support virulence. For example, this is the case for the genes *tpsA* and *tpsB*, coding for trehalose-6-phosphate synthase of *A. fumigatus*. In *C. albicans* and *C. neoformans* [59–62], these genes mediate stress response and virulence, but in *A. fumigatus* their inactivation actually triggers cell wall alterations that lead to enhanced immune evasion and hypervirulence *in vivo* [63]. FleA is a lectin of *A. fumigatus* that binds fucosylated structures, which are abundant in the glycan coats of many plant and animal proteins. Kerr et al. [64] showed that FleA of *A. fumigatus* conidia binds to airway mucins which allow macrophages to effectively phagocytose them. Deletion of FleA accordingly reduces phagocytosis, and mice infected with *fleA*Δ conidia develop more severe pneumonia and invasive aspergillosis than with wild-type conidia. It is not certain why *A. fumigatus* and other pathogenic fungi have evolved to express such a protein, but it is thought that FleA can help to grow on carbohydrate-rich surfaces [64]. Therefore, the FleA lectin acts as an antivirulence factor during *A. fumigatus* infections, and its loss can be advantageous for *A. fumigatus* growing on human mucosae. This concept also explains why antivirulence factors are still present in pathogenic fungi of mammals. As most of them exist in the environment or as commensals most of the time, these genes are adaptive for the majority of possible niches. Only when they enter the host, the new selective forces can work to inactivate these genes. A closer look at clinical isolates from severely ill patients, in comparison with commensal or environmental strains would most likely reveal more potential antivirulence genes.

## The concept and examples of effectors and avirulence genes in human pathogenic fungi

**Figure 2.**
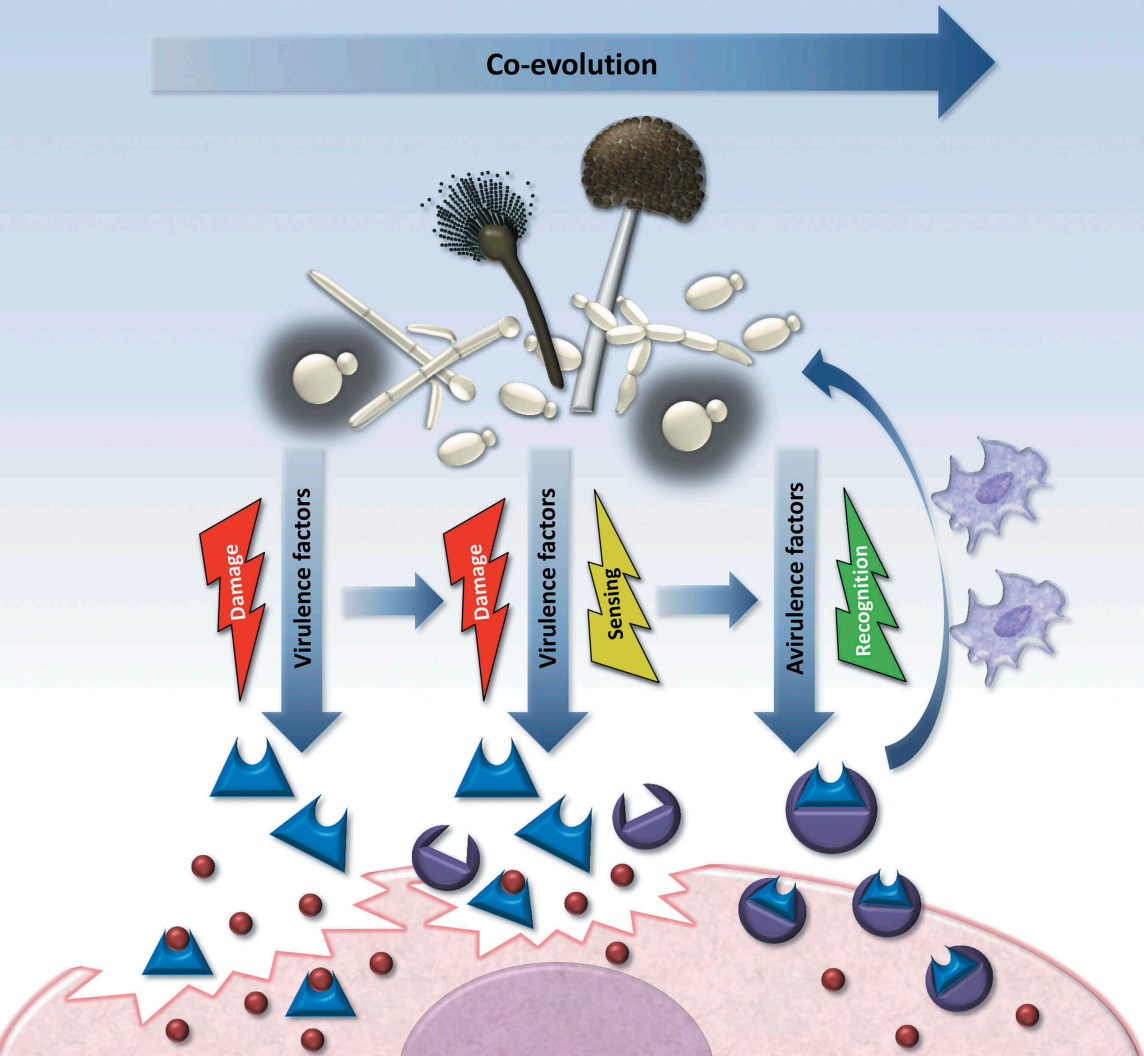
Evolution of a virulence factor to an avirulence factor as a result of antagonistic co-evolution. A virulence factor confers the pathogen with an adaptive advantage within the host environment, which allows the infection to progress. However, frequent host–pathogen interactions act as a selection pressure on the host side to develop a specific defense response. As a result of this co-evolution the host can develop receptors that specifically recognize the pathogen’s virulence factor and trigger a specific (immune) response that counteracts and thereby abolishes the pathogen’s virulence. Note that new avirulence factor can still serve as a virulence factor in susceptible hosts that have not yet developed the specific response.

As described above, virulence factors contribute to the development of host damage and thus disease. However, as damage-associated factors, they can also trigger recognition by the host. This can happen either by a damage-mediated host response [18,19,25] or at an earlier stage due to antagonistic evolution (Figure 2): if the host has “learned” to recognize the virulence factor and raise specific defense mechanisms against it even before damage commences. Common examples of such a coevolution are found in fungal pathogens of plants [20,21,65,66]. These fungi rely on effectors, which are generally secreted molecules that modulate the interaction between the fungus and the host at different steps of infection [20]. Not unlike the mammalian immune system, plants have developed a multi-layered defense against fungal pathogens. The initial, general response is triggered by microbe-associated molecular patterns and comprises unspecific antimicrobial compounds [20]. Plant-pathogenic fungi overcome this barrier with secreted effectors, which can suppress the host immune response, or manipulate host cell physiology to support fungal survival. For example, *Cladosporium fulvum* is a biotrophic fungal pathogen that causes leaf mold of tomato (*Solanum lycopersicum*). In the host, it releases the effector Ecp6 to sequesters chitin oligosaccharides detached from its own cell walls, and thereby prevents a triggering of the host immunity [67]. The corn smut agent, *Ustilago maydis*, secretes high amounts of the chorismate mutase Cmu1 during plant colonization. This enzyme reduces the levels of salicylic acid precursors and thus neutralizes salicylic acid-induced immune responses [68].

To counterattack the suppressing action of the fungal effectors on the first line of plant defense, plants have evolved a second layer of defense, termed effector-triggered immunity, through production of receptors that specifically recognize the pathogen virulence effectors. Thus, plant protein receptors detect the fungal effectors and trigger a hypersensitive response consisting on localized cell death to stop the propagation of the infection, rendering the plant resistant. Since this makes the fungi carriers of this specific effector avirulent in this specific plant species, these fungal effector-encoding genes are known as avirulence genes (*AVR*) (Figure 1). It is noteworthy that they contribute to either virulence or avirulence, depending on the present of a corresponding receptor gene (*R*) in plant host [20,69]. This host–pathogen interaction is known as gene-for-gene relationship, in which the host possesses a specific gene (*R*) whose product targets a certain virulence effector encoded by another specific gene of the pathogen (*AVR*) [70].

The effector Avr2 is present in *C. fulvum* and selectively binds and inhibits plant proteases involved in basal defense. In resistant plants, however, the receptor Cf-2 recognizes the antivirulence-protease and activates a hypersensitive response [71,72]. Such complex *R-AVR* interactions likely result from the long coevolution between plants and their pathogens: Effector evolution is therefore a trade-off between escaping detection and optimizing the virulence-related functions. In the long term, pathogen ﬁtness may rely on the continuous emergence of novel effectors as replacement for those that are detected by the host. This implicates a strong evolutionary pressure on effectors, and genome-wide analyses of plant pathogenic fungi have in fact demonstrated a higher degree of positive selection in genes encoding secreted proteins compared with genes encoding non-secreted proteins [20,73–76].

With this in mind, can we expect to find similar mechanisms in human-fungal pathogens? Among the few species of pathogenic fungi of humans that live as a commensal within or in close contact with the host are *Candida* spp., for which we would expect the strongest signs of co-evolution [3]. The majority of human pathogenic fungi are opportunists that are adapted to environmental niches; however, effectors adapted to distinct niches may still confer adaptive advantages within the human body. This concept, where fungi gain pathogenic potential in environmental niches is known as “(environmental) virulence schools”[3]. It is thought that environmental fungi evolve to adhere to surfaces, form biofilms, compete with bacteria, acquire all necessary nutrients and deal with changes in temperature, pH, osmolarity and other physical stresses, all relevant factors for survival in the human host [10,12,77–79]. Moreover, it is known that *A. fumigatus, C. neoformans, Coccidioides* spp. and *H. capsulatum* are facing soil amoeba [3,15,80], which share many characteristics with human phagocytes [15]. Thus, the virulence attributes displayed by these fungi pathogens may be advantageous to defend both, against environmental phagocytes and phagocytes from animal hosts, and therefore, the study of the interaction of environmental fungi and amoeba may uncover potential virulence and avirulence genes.

Effectors of plant pathogens can act in the cytoplasm (when directly delivered *via* sophisticated systems – intracellular effectors) or the apoplast (the extracytoplasmic space – extracellular effectors) of plants to modulate the immune responses. Examples of analogous protein functions which act extracellularly can be found in both, commensal and environmental human pathogenic fungi. Here they are involved in host immune evasion or modulation in favor of the pathogen [14,81,82]. For example, the human complement system, an important contributor to innate immunity, is a common target for manipulation by fungal pathogens [83–85]. *C. albicans* and *A. fumigatus* express proteins that can bind or inactivate complement proteins: Secreted and cell surface-localized Pra1 [86–88], Hgt1 [89], Gdp2 [90], and Gmp1 [91] of *C. albicans* can bind complement evasion-mediating human compounds like factor H, plasminogen, and others; the *C. albicans* Sap-family of aspartic proteases and the Alp1 protease of *Aspergillus* spp. can degrade the effector components of the terminal complement complex [92–94]. *Aspergillus* species also synthesize a soluble complement inhibitor, which prevents complement activation and opsonization [83]. Similarly, the secreted protein App1 of *C. neoformans* can inhibit complement receptor-mediated phagocytosis by macrophages [84,95]. *A. fumigatus* releases the metabolite gliotoxin which is immunosuppressive and able to induce apoptosis of monocytes and macrophages [96–99]. Lastly, *C. albicans* Sap proteases have been shown to have the potential to degrade host antibodies [100,101] and the two surface-bound member, Sap9 and Sap10, can inactivate antimicrobial peptides (AMP) released by the host [102]. Importantly, many of these proteins are themselves potent triggers of host responses: Sap proteases trigger inflammation [103] and Pra1 derives its name from being a pH-regulated antigen of *C. albicans* [104].

An example reminiscent of the gene-for-gene mechanism in plant pathogenesis is the recently discovered Melanin-sensing C-type Lectin receptor (MelLec) involved in immunity against *A. fumigatus* [105]. MelLec recognizes the naphthalene-diol unit of *A. fumigatus* melanin, one of the most important virulence factors of this fungus [106] . In mouse models and in humans this receptor seems to be required for resistance against *Aspergillus* infections [105]. The existence of a very specific receptor in the host for a fungal virulence factor thus renders the pathogen avirulent in otherwise healthy hosts.

Further examples of potential avirulence genes can be found in the most pathogenic species of the *Candida* group: *C. albicans*. This fungus possesses a hyphae-associated gene, *DUR31*, which not only contributes to epithelial damage but is also required in multiple stages of candidiasis, including surviving attack by human neutrophils and mediating endothelial damage [107]. It is thus required for full virulence *in vivo*. However, Dur31 also transports histatin 5 (Hst 5), a highly cytotoxic human AMP, into the fungal cell, thereby committing a suicide-like process [107]. Therefore, Dur31 is an indispensable protein for the normal progress of infection, but the host has “learned” to take advantage of it against the pathogen. This is not unlike the response of plants to certain fungal effectors (avirulence effectors) [108], and can be considered another potential example for a gene-for-gene relationship (in that case, histatin 5 exploiting the presence of Dur31). The heat shock protein Ssa1, present on the surface of hyphae, but not yeasts, of *C. albicans* is another example. It acts as an invasin, and a *ssa1*Δ/Δ mutant shows attenuated virulence in mouse models of both disseminated and oropharyngeal candidiasis [109]. However, Ssa1 also facilitates transport of the antimicrobial peptides Hst 5 and β-defensins, enabling their activity inside the fungal cell [110,111]. Furthermore, the fungal toxin candidalysin is considered an important virulence factor of *C. albicans* and one of the very few “classical virulence factors” of human pathogenic fungi [112,113], which directly damages host cells and allows fungal hyphae to cross the epithelial barriers [113,114]. However, candidalysin can also activate the epithelial “danger response” pathway [113,115]. This alerts the host to the presence of invasive, toxin-producing hyphae and induces protective immune responses [113]. In fact, it was shown that oral epithelial cells orchestrate innate Type 17 responses to *C. albicans* hyphae through candidalysin [116]. Thus, because of the dual property of this toxin as a damage agent and an activator of the immune system, it may be considered an avirulence factor in the immune competent host (and like its plant counterparts, a virulence factor in the susceptible host). Supporting this argument, two further recent studies have shown more dual, virulence and avirulence traits of candidalysin: on one hand, this fungal toxin contributes to killing of macrophages, but also activates the inflammasome, with production of the pro-inflammatory cytokine IL-1β and recruitment of other immune cells to the site of infection [117]. Similarly, it also promotes antifungal immunity in the central nervous system by activating the production of neutrophil-recruiting IL-1b and CXCL1 in microglia [118] . However, whether candidalysin acts as a virulence or an avirulence factor also depends on the site and tissue of infections: during vaginal infections, candidalysin triggers an immune response, which is associated with immunopathology and thus diseases [119]. Thus, virulence has not only to consider the pathogen and the host but also to consider the specificities of a particular host niche.

## Conclusion

Is it helpful to argue in favor of applying the concept of antivirulence and avirulence genes to human pathogenic fungi, as we did in this review? We think yes, as the way we name and label biological phenomena determine to a great degree how we think of them. Not trying to fit everything in these traditional boxes, or maybe taking a different point of view and accepting new paradigms (or established ones from other fields) can thus help to ask new questions and bring science forward. For example, while the host-mediated, local destruction of tissue in response to fungal growth is called a hypersensitive response in plants (and considered beneficial in limiting spread of the pathogen), it is seen as a damaging, overshooting immunopathology in mammals. In that manner, plant-pathogen effectors which elicit a hypersensitive response are treated as avirulence factors, while, e.g. candidalysin, when it causes localized immune-mediated, vaginal tissue damage [119], is seen as a virulence factor. In both cases, the fungal pathogens have the capacity for systemic infection of susceptible host, and the same immune mechanisms that stop their spread also lead to local tissue damage. This comparison admittedly glosses over many of the intricacies of the respective host–pathogen interactions, but it can be very helpful for researchers in fungal pathology on occasion to change their perspective and maybe the nomenclature they hold dear, in order to correctly ask the next important research question.

There are many possible benefits: The identification of antivirulence and avirulence genes in more pathogenic fungi would help us to comprehend the emergence and evolution of pathogenesis. As in bacterial pathogens, the reintroduction of functional antivirulence genes in their respective pathogens may allow to create vaccine candidates against fungal infections [120]. Moreover, search and identification of fungal effectors that trigger host immune responses would also uncover possible new virulence factors and potential antifungal targets. Thinking in terms of antivirulence and avirulence in the field of human-fungal pathogens will thereby, we hope, help to achieve a better understanding of fungal virulence and disease.
